# Blood-based gene expression profiling in castrate-resistant prostate cancer

**DOI:** 10.1186/s12916-015-0463-8

**Published:** 2015-09-14

**Authors:** Mitchell E. Gross

**Affiliations:** Center for Applied Molecular Medicine, Keck School of Medicine, University of Southern California, 9033 Wilshire Boulevard, Suite 300, Beverly Hills, CA 90211 USA

**Keywords:** Castrate-resistant prostate cancer, Gene expression profiling, Prognostic biomarker

## Abstract

Castrate-resistant prostate cancer (CRPC), the most life-threatening form of prostate cancer, has recently been the focus of many successful new treatments. Contemporary trials highlight the heterogeneous prognosis of CRPC as overall survival times vary greatly across different patient sub-groups. As presented in *BMC Medicine*, Wang et al. identify a blood-based prognostic signature in CRPC. Their approach is notable for discovery and validation of a four-gene model based on a whole-blood expression signature sampled from three distinct clinical cohorts. Further, the marker selection process incorporates an understanding of biological pathways expressed in myeloid or lymphoid cells which may provide some insight into host-tumor interactions as reflected in the peripheral blood. While the study includes a multivariate analysis accounting for many important clinical variables, larger datasets with more complete clinical information and sufficient follow-up are needed to confirm the independent significance of the four-gene expression model in a way which may better inform the care of CRPC patients.

Please see related article: http://www.biomedcentral.com/1741-7015/13/201.

## Introduction

Prostate cancer is a heterogeneous disease. At diagnosis, clinical and pathologic features, such as prostate specific antigen level, pathologic Gleason grade, and tumor stage, are used to inform clinical decision making [1]. For some patients, prognostic factors are interpreted as favorable (and the disease is projected to proceed slowly) that no active therapy is pursued (“watchful waiting” or “active surveillance”). Other patients are diagnosed with a highly aggressive form of prostate cancer which is either metastatic at diagnosis or recurs after local therapy (typically surgery or radiation). Androgen-deprivation therapy is the mainstay of therapy for metastatic/recurrent prostate cancer. However, cancer can progress despite medical or surgical castration to castrate-resistant prostate cancer (CRPC).

Heterogeneity is also noted among patients with advanced and recurrent forms of prostate cancer. While CRPC is the most dangerous, and often fatal, form of prostate cancer, median survival times reported for patients treated with highly-active androgen targeting agents in chemotherapy-naïve CRPC are well over 24 months in most contemporary trials [2, 3]. In contrast, active treatment with a bone-targeted radiopharmaceutical (Radium-223) in chemotherapy-naïve patients in a recent trial reported a median survival of only 14 months in the active treatment arm [4]. While the later trial focused on subjects who were deemed not candidates for cytotoxic chemotherapy based on physician discretion or patient preference, the inclusion criteria between these trials were generally overlapping. Thus, there is an opportunity to explore readily-obtained, objective biomarkers to more accurately determine prognosis in CRPC.

Wang et al. [5] sought to define a prognostic model in CRPC based on analysis of a whole-blood RNA expression signature. Starting with expression data generated in a publicly available data set [6], the authors identified co-expression modules for gene sets which discriminate based on prognosis. When grouped and compared with other annotated gene expression signatures, the “up modules” were enriched for genes associated with erythroid and myeloid cells, while “down modules” were enriched for genes associated with B and T lymphocytes. Genes representing the greatest differences in expression levels between good and poor risk groups were used to develop a prognostic model using a Bayesian learning algorithm. A four-gene model incorporating MCM2, PROS1, CD22, and TMEM66 performed best in cross-validation tests and was selected for further assessment. The model was tested in two separate cohorts of patients totaling 90 patients and was shown to add statistically significant prognostic information to standard clinical prognostic variables.

Whole blood expression signatures have been examined in relation to prognosis for prostate and other forms of cancer [6–9]. Relatively unique in the Wang et al. [5] study is the overlay of functional information, including cell-type-specific expression patterns and pathway information, with gene expression. Thus, it is interesting to note that up-regulation of myeloid-associated genes was associated with a worse prognosis consistent with the finding that increases in tumor-associated macrophages confer a worse prognosis [10, 11]. The increased expression of myeloid relative to lymphoid markers in poor risk patients is also consistent with other studies focusing on the neutrophil to lymphocyte ratio, obtained with a standard peripheral blood count, as an independent prognostic factor in CRPC [12–15]. Immune activation in relation to prostate cancer is an area of great interest. Many studies have sought to identify association and mechanisms between specific immune cells with the development and progression of prostate cancer [16]. This focus is further heightened by the use of an immuno-therapy (sipuleucel-T) for patients with CRPC [17]. It is interesting to consider how approaches like whole-blood expression signatures can help characterize the nature of immune-activation directed against cancer in hopes of more specifically stimulating tumor-immunity with therapeutic intent.

## Conclusion

The therapeutic landscape of CRPC has changed rapidly in the last few years as entirely new classes of therapies (high-potency androgen targeting agents, bone-targeted radiopharmaceuticals, immunotherapy) have been added to conventional hormonal and chemotherapy agents approved for CRPC, often with overlapping indications. While improved prognostic markers are significant, there is an even greater need to find biomarkers to select patients for specific therapies. The study by Wang et al. [5] suggests that a biologically-informed approach to the analysis of blood-based expression signatures may be one approach towards this goal.
